# Resistance, virulence, and population genomic structure of avian pathogenic *Escherichia coli* from poultry airsacculitis in South Africa

**DOI:** 10.1016/j.psj.2026.107885

**Published:** 2026-10-05

**Authors:** Zhuohao Wang, Naima Ameris Guthauser, David Gerber, Vincent Perreten

**Affiliations:** a Division of Molecular Bacterial Epidemiology & Infectious Diseases, Institute of Veterinary Bacteriology, Vetsuisse Faculty, University of Bern, Bern, Switzerland; b V-Tech (Pty) Ltd, Midrand, South Africa; c Dunevax Biotech (Pty) Ltd, Windhoek, Namibia

**Keywords:** APEC, Genomics, Phylogeny, Antimicrobial resistance, Virulence

## Abstract

A total of 109 avian pathogenic *Escherichia coli* (APEC) were isolated from poultry with airsacculitis in 21 different farms in South Africa between June 2021 and January 2022. All isolates were sequenced using Illumina technology and one representative strain from each detected sequence type (ST) was sequenced using Oxford Nanopore technology (ONT) to obtain complete genomes for single nucleotide polymorphism (SNP) analysis. Whole genome sequences (WGS) were used for molecular serotyping, sequence typing, virulence and antimicrobial resistance genes detection, core genome multilocus sequence typing (cgMLST)-based phylogeny, and cgSNP. The MICs of 15 antibiotics were tested by broth microdilution. A total of 21 serotypes and 18 STs were detected, with O78:H4-ST117 (n=29), O36:H5-ST5764 (n=10), and O78:H4-ST15549 (n=9) being the most common. Comparative genomic analyses revealed that some strains within the same serotype-ST combinations were genetically closely related (<20 cgSNPs) and were isolated within the same farm and across different farms. Each serotype-ST combination exhibited specific virulence and resistance profiles. Of 172 genes covering 51 virulence factors identified, 15 of them were shared by all strains. Resistance to ampicillin, ciprofloxacin, nalidixic acid, tetracycline, and trimethoprim was most frequent, each occurring in over 50% of the strains. Multidrug resistance to 3–9 antimicrobials was observed in 58.7% of the isolates. Antimicrobial resistance phenotype was associated with the presence of acquired resistance genes and mutations.

This study provided an in-depth WGS-based characterization of genomic features revealing a high number of different virulence and antimicrobial resistance genes, also highlighting a possible vertical and horizontal transmission of several APEC lineages in the poultry chain in South Africa.

## Introduction

*Escherichia coli*, a member of Enterobacterales, is part of the normal bacterial flora of poultry. A weakened immune system and consequently reduced resistance to diseases can enable opportunistic pathogenic *E. coli* to spread to the respiratory tract and enter the bloodstream, causing a range of systemic symptoms in avian species, including airsacculitis, perihepatitis, pericarditis, and septicemia (Jamali et al., 2024). The disease associated with these symptoms in poultry is known as avian colibacillosis and is caused by avian pathogenic *E. coli* (APEC). APEC is a major cause of mortality and morbidity in poultry leading to substantial economic losses in the poultry industry worldwide (Kathayat et al., 2021; Joseph et al., 2023).

APEC harbors diverse virulence factors like adhesins, invasins, iron acquisition systems, and the occurrence of APEC-specific toxins in human extraintestinal pathogenic *E. coli* (ExPEC) suggests a zoonotic potential (Hu et al., 2022). Although numerous serotypes and sequence types (STs) have been reported in APEC, certain predominant lineages appear to be particularly successful in causing disease in poultry (Dho-Moulin and Fairbrother, 1999; Jeong et al., 2012; Kim et al., 2020; Nawaz et al., 2024). Serogroups O1, O2, and O78 represent the three most prevalent serogroups and are associated with up to 80% of APEC infections in poultry (Cloud et al., 1985; Dozois et al., 1992; Khairullah et al., 2024; Nawaz et al., 2024). The frequent occurrence of a limited number of STs, such as ST10, ST48, ST95, and ST117 (Dissanayake et al., 2014; Ronco et al., 2017; Azam et al., 2020; Mehat et al., 2021), further suggests that strains of certain genetic lineages may contribute to the major cases of infection. Nevertheless, the emergence and circulation of diverse and new APEC lineages complicate the control of colibacillosis, which relies on vaccination that offers targeted protection and the use of antibiotics as a complementary measure (Watts and Wigley, 2024). Two vaccines (live-attenuated APEC O78 ΔaroA Poulvac® *E. coli* vaccine and inactivated Nobilis® *E. coli* vaccine containing F11 fimbrial and FT flagellar antigens) are commercially available for use in chickens and have been reported to provide limited protection against APEC (Gregersen et al., 2010; Ghunaim et al., 2014; Joseph et al., 2023). Antimicrobials such as tetracyclines, sulfonamides, and aminoglycosides are widely used, raising concerns regarding the rising level of antimicrobial resistance among APEC worldwide (Jamali et al., 2024).

In this study, we used a WGS-based approach to characterize APEC isolates associated with airsacculitis across different poultry farms in South Africa and investigate their genomic diversity, virulence-associated genes, and antimicrobial resistance determinants.

## Materials and methods

### Sample collection, isolation and identification of strains

During June 2021 to January 2022, 109 samples were collected from airsacculitis lesions using sterile cotton swabs during postmortem examinations of broilers and breeders with ages ranging from 1 day to 42 days for broilers to up to 62 weeks for breeders) in 21 different farms in South Africa. It is unknown whether the farms used vaccination against APEC. The samples were spread onto chromogenic tryptone bile X-glucuronide agar (AEC-Amersham) and incubated at 37°C for 24 h. Colonies were then cultured on trypticase soy agar with 5% sheep blood (TSA-S) (Becton Dickinson) to obtain pure cultures. Species identification was performed by Matrix-Assisted-Laser-Desorption/Ionization-Time-Of-Flight-Mass-Spectometry (MALDI-TOF MS) according to manufacturer’s standard protocol (Bruker Daltonics GmbH, Bremen, Germany). The identification as *E. coli* was considered reliable only when the score was above 2.00. The isolates were frozen at -80°C in 30% glycerol stocks.

### Whole genome sequencing, genome assembly and annotation

WGS was performed for all the 109 *E. coli* isolates using Illumina technology (Illumina). DNA library was prepared using the Nextera^TM^ DNA flex microbial colony extraction protocol and the Illumina® DNA Prep (M) Tagmentation kit (Illumina). All libraries were sequenced on an Illumina NovaSeq 6000 system (S4 flow cell, 300 cycles) at the Next Generation Sequencing Platform, Institute of Genetics, University of Bern. One strain from each ST group was also sequenced by Oxford Nanopore Technology (ONT) to be used as a reference sequence for single nucleotide polymorphism (SNP) calling. Strain EC34195 isolated from a commercially available live-attenuated *E. coli* O78 *aroA*-deleted vaccine (Poulvac®) was also sequenced with Illumina technology for genomic comparison. The Illumina reads were *de novo* assembled with Unicycler (Illumina-only; v.0.5.0) (Wick et al., 2017) using Trimmomatic (Bolger et al., 2014)-trimmed paired-end short reads. The complete genome assembly of each representative ST were obtained by hybrid assembly of short Illumina reads and long ONT reads using Unicycler (Hybrid; v.0.5.0) (Wick et al., 2017), Trycycler (Wick et al., 2021), Medaka (https://github.com/nanoporetech/medaka), and Polypolish (Wick and Holt, 2022), to obtain complete high-quality circular contigs. ORFs of all the assembled genomes were identified and annotated by using PGAP v.6.8 upon submission to the NCBI GenBank (Tatusova et al., 2016).

The Illumina and ONT raw reads were deposited into the Sequence Read Archive (SRA) database of the National Center for Biotechnology Information (NCBI) GenBank database under the BioProject no. PRJNA1117783. The complete genome of 18 strains representing the 18 identified STs were deposited into the NCBI database under the BioProject no. PRJNA1117783 with corresponding BioSample and GenBank accession numbers, namely SAMN41579897 (CP178657–CP178659), SAMN41579871 (CP178630–CP178634), SAMN41579872 (CP178635–CP178642), SAMN41579880 (CP178643–CP178648), SAMN41579881 (CP178649–CP178650), SAMN41579890 (CP178651–CP178654), SAMN41579893 (CP178655–CP178656), SAMN41579899 (CP178660–CP178662), SAMN41579901 (CP178663–CP178667), SAMN41579907 (CP178712–CP178715), SAMN41579908 (CP178668–CP178670), SAMN41579913 (CP178671–CP178675), SAMN41579927 (CP178676–CP178682), SAMN41579938 (CP178683–CP178690), SAMN41579944 (CP178691–CP178694), SAMN41579951 (CP178695–CP178701), SAMN41579967 (CP178702–CP178707), SAMN41579968 (CP178708–CP178711). The accession numbers to sequence data and associated specific genomic characteristics of each strain are available in Table S1. Assembly quality metrics of the 18 complete genomes, including genome size, GC content, number of contigs, N50, sequencing coverage, and genome assembly accession numbers are provided in Table S2.

### WGS-based molecular characterization

Molecular serotyping based on the O and H antigens (Joensen et al., 2015), multilocus sequence typing (MLST) (Maiden et al., 1998), and antibiotic resistance gene detection based on NCBI AMRFinderPlus (https://www.ncbi.nlm.nih.gov/pathogens/antimicrobial-resistance/AMRFinder/) (Feldgarden et al., 2019) were performed using Ridom SeqSphere+ (v.9.0, Ridom GmbH, Münster, Germany). ResFinder (v.4.5.0) (Bortolaia et al., 2020) was also used to validate the accuracy of AMR genes detection. New STs were assigned through EnteroBase (https://enterobase.warwick.ac.uk/). Phylogroups including A, B1, C, D, E, and F were assigned using ClermonTyping, based on the method described by Clermont et al. (https://github.com/iame-researchCenter/ClermonTyping) (Beghain et al., 2018; Clermont et al., 2019). Virulence genes were detected using ABRicate with the VFDB database (Chen et al., 2005). To detect the 10 key virulence-associated genes recently highlighted in APEC (*iss, tsh, iroN, ompT, iutA, cvaC, hlyF, iucD, papG*, and *papC*) (Ovi et al., 2023), *iss, ompT, cvaC*, and *hlyF*, which were absent from the VFDB database, were additionally screened using NCBI BLAST+ v.2.5.0 (Camacho et al., 2009). The reference sequences of these genes were extracted from plasmid pVTB777v-1 (GenBank accession number CP178635.1). A gene was considered present when both sequence identity and query coverage exceeded 90%.

### Comparative genomic analysis

Genome-wide gene-by-gene alleles of each genome were called by Ridom SeqSphere+ (v.9.0, Ridom GmbH, Münster, Germany) using paired-end short-reads with *E. coli* cgMLST Scheme (https://www.cgmlst.org/ncs/schema/Ecoli540/; last change: Apr 15, 2024). Complex type (CT) was called and phylogenetic tree was constructed based on these allele combinations and visualized by iTOLv6 (Letunic and Bork, 2021). Core SNP analysis was determined using Snippy v.4.6.0 and snippy-multi script (https://github.com/tseemann/snippy). The 15 strains representing each of the 15 different STs detected more than once were used as reference sequences for cgSNP analysis. The cgSNP distance matrix was calculated by snp-dists v.0.8.2 (https://github.com/tseemann/snp-dists) and was visualized by TBtools (Chen et al., 2020). Isolates differing by < 20 cgSNPs were considered as genetically closely related indicating potential recent transmission or cross-source linkage (Sealey et al., 2026).

### Antimicrobial susceptibility testing

The minimum inhibitory concentration (MIC) of all isolates were determined by broth microdilution techniques in Mueller-Hinton Broth using Sensititre^TM^ EUVSEC3 plates following manufacturer’s protocols (ThermoFisher) and according to the EUCAST guidelines (https://www.eucast.org/). The following 15 antimicrobials were tested: ampicillin, meropenem, ciprofloxacin, azithromycin, amikacin, gentamicin, tigecycline, ceftazidime, cefotaxime, chloramphenicol, colistin, nalidixic acid, tetracycline, trimethoprim, and sulfamethoxazole. MICs were interpreted using the resistance breakpoints from EUCAST (The European Committee on Antimicrobial Susceptibility Testing, 2026), except for nalidixic acid, tetracycline, and sulfamethoxazole, for which breakpoints from CLSI were used (Clinical and Laboratory Standards Institute, 2026). As no breakpoint exists for azithromycin, an MIC of > 16µg/mL was tentatively used as a breakpoint as suggested by Gomes et al. (Gomes et al., 2019).

## Results

### WGS-based molecular epidemiological characterization

***Molecular Typing***. Molecular analysis of the 109 APEC revealed 21 serotypes, 18 STs, and 6 phylogroups, with serotypes O78:H4 (n=40), O36:H5 (n=10), O5:H10 (n=7), sequence types ST117 (n=31), ST23 (n=11), ST93 (n=10), ST5764 (n=10), ST15549 (n=9), and phylogroups G (n=31), A (n=24), C (n=20) being the most frequent (Fig. 1). An association between serotype and ST was observed, with 14 STs each exclusively linked to a specific serotype (Fig. 1). The remaining four STs (ST117, ST23, ST93, and ST69) were associated with two to three serotypes (O78:H4/O36:H4/O36:U-ST117; O8:H9/O78:H9/O78:H4-ST23; O5:H10/O5:H40-ST93; O15:H18/O15:H6-ST69). On the other hand, two serotypes were associated with two to three different STs (U:H5-ST140/ST355 and O78:H4-ST117/ST15549/ST23). The most frequent serotype-ST-phylogroup combinations consist of O78:H4-ST117-G (n=29), O36:H5-ST5764-A (n=10), O78:H4-ST15549-C (n=9), O5:H10-ST93-A (n=7), U:H5-ST140-B2 (n=7), O8:H9-ST23-C (n=6), O145:H10-ST1049-B1 (n=6), and U:H1-ST429-B2 (n=6) (Fig. 1).

**Fig. 1:**
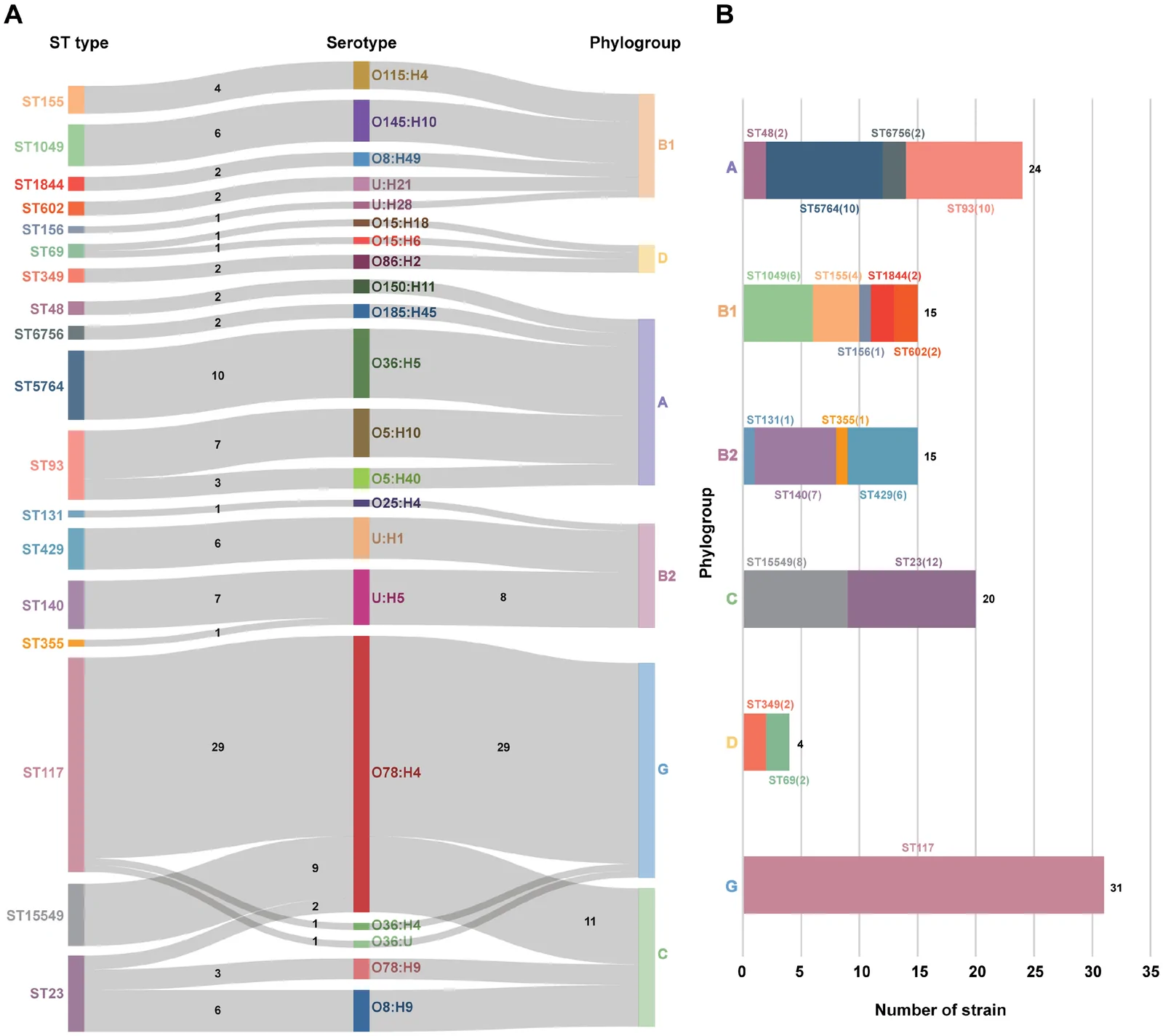
Correlation between sequence types (ST), serotypes and phylogroups (A) and number of ST present in each phylogroups (B) of 109 APEC from poultry airsacculitis in South Africa. The Sankey diagram and stacked bar in this figure were generated by R v.4.1.2 and Microsoft Excel 2019 v.1808, respectively. The final visualization was further refined using Adobe Illustrator v.24.0.

***cgMLST- and SNP-based Comparative Analysis.*** Mapping serotype, ST, and farm origin onto the cgMLST-based phylogenetic tree showed that strains belonging to the same phylogroup clustered within the same clades (Fig. 2). To better determine genetic relatedness between strains and identify lineages within the same ST, cgSNP distances within each ST were also determined and shown in Fig. 2. SNP analysis revealed that genetically closely related strains differing by < 20 cgSNPs were present within groups of strains belonging to the same serotype-ST combination. Using this threshold, some strains formed specific lineages within serotype-ST combinations of O115:H4-ST155 (0 cgSNP within each of the two lineages), O5:H10-ST93 (2–7 cgSNPs within each of the two lineages), O8:H9-ST23 (0–11 cgSNP within each of the two lineages), U:H5-ST140 (0–1 cgSNP within each of the three lineages), O36:H5-ST5764 (0–4 cgSNP within each of the three lineages), O78:H9-ST23 (0–2 cgSNP within each of the two lineages). Larger cgSNP distances were observed for strains within O5:H10-ST93 (584–591 cgSNPs), O36:H5-ST5764 (1920–1921 cgSNPs), U:H5-ST140 (525–574 cgSNPs), U:H21-ST602 (739 cgSNPs). As expected, strains of different serotypes within the same ST exhibited substantial cgSNP differences. For instance, 5277–5809 cgSNPs were observed between strains of O78:H4-ST117 and O36-ST117, 3787 cgSNPs between strains of O36:H4-ST117 and O36:U-ST117, 6939–6942 cgSNPs between strains of O5:H40-ST93 and O5:H10-ST93, 2813–2830 cgSNPs between strains of O78:H4-ST23 and O8:H9-ST23, and 1378–1401 cgSNPs between strains of O8:H9-ST23 and O78:H9-ST23. Notably, the O8:H9-ST23 strains shared the same serotype-ST combination as vaccine strain EC34195 but differed from it by 285–288 cgSNPs. They also contained a complete *aroA* gene, which is deleted in EC34195, indicating that the O8:H9-ST23 field strains were not related to vaccine strain EC34195.

**Fig. 2:**
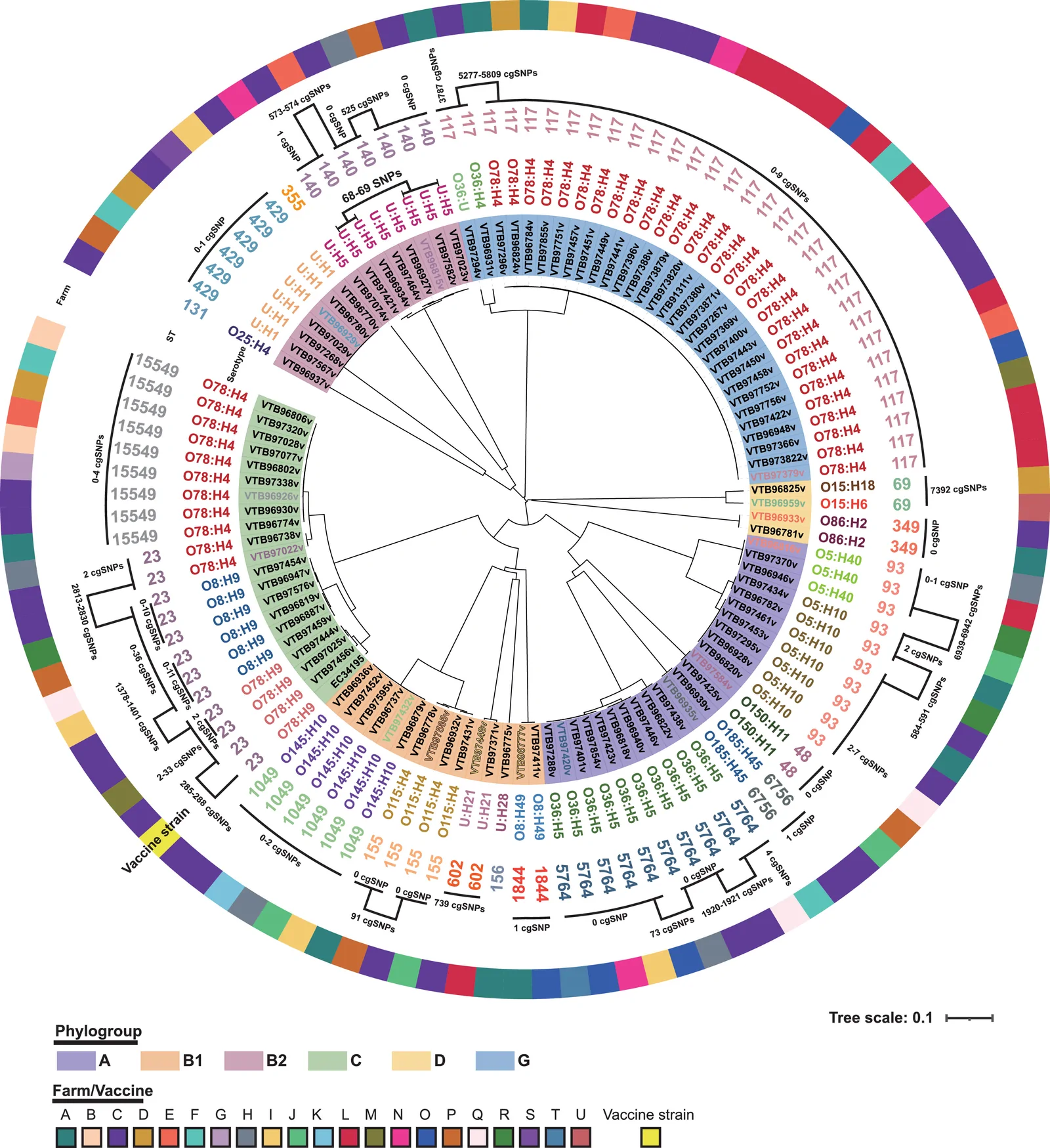
cgMLST-based phylogenetic tree of 109 APEC strains recovered from poultry with airsacculitis in South Africa. Vaccine strain EC34195 was included for genomic comparison. Clermont phylogroups are differentiated with different background colors. The serotype, sequence type (ST), and farms are indicated alongside the tree. For cgSNP analysis, one representative strain from each ST was selected as the reference genome and is indicated using the same color as the corresponding ST. The range of pairwise cgSNP distances within each ST was also shown. The phylogenetic tree was generated by Ridom SeqSphere+ (v.9.0, Ridom GmbH, Münster, Germany), annotated and visualized using iTOLv6, and further refined using Adobe Illustrator v.24.0.

*Distribution of APEC in Broilers and Breeders Within and Between Farms:* Genetically related strains were identified within the same farms as well as across different farms (Fig. 2, Fig. 3). Among the most frequent serotype-ST combinations, genetically closely related strains exhibiting <20 cgSNPs, indicative of recent transmission, were present across different farms. For instance, 29 strains of O78:H4-ST117 differing by 0–9 cgSNPs were detected in 10 different farms, 9 strains of O78:H4-ST15549 (0–4 cgSNPs) in 7 farms, 6 strains of U:H1-ST429 (0–1 cgSNP) in 6 farms, 6 strains of O145:H10-ST1049 (0–2 cgSNPs) in 5 farms. On the other hand, different lineages were also found within the same farms (Fig. 3). Among the farms with the largest numbers of samples (Farm A, C, L), 21 different lineages (< 20 cgSNPs) covering 17 different serotype-ST combinations were found among 31 strains in Farm C, 9 lineages of 8 serotype-STs among 10 strains in Farm A, 3 lineages were observed in 13 Farm L strains and the majority of them belonged to lineage O78:H4-ST117 (n=11). The presence of genetically closely related strains in both broilers and breeders within the same farms as well as across different farms suggest possible horizontal and vertical transmission of APEC.

**Fig. 3:**
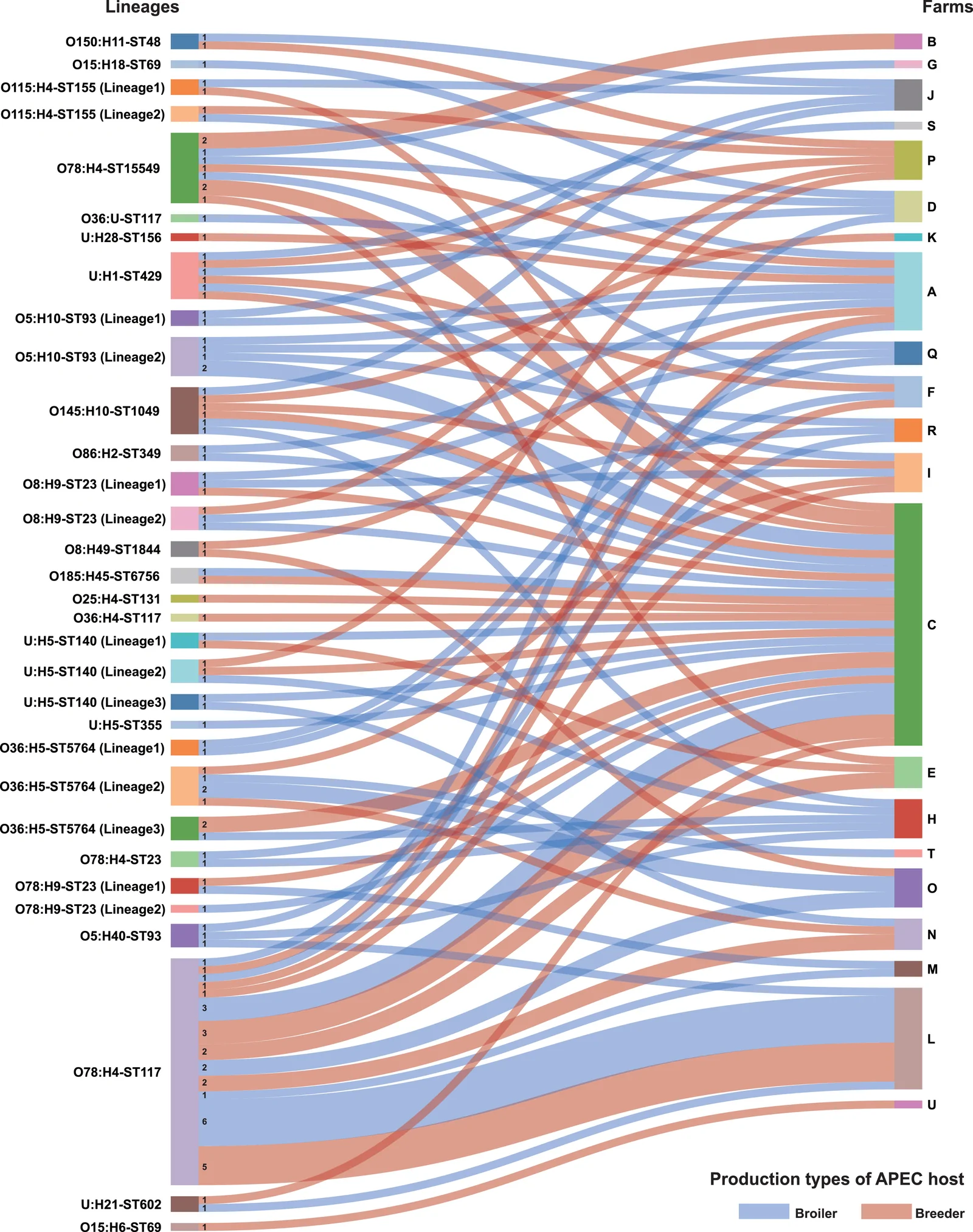
Correlation and association between serotype-ST combinations of lineages (<20 cgSNPs), farms (A to U) and broilers and breeders in 109 APEC strains from poultry airsacculitis in South Africa. Broilers are indicated by blue lines and breeders are indicated by pink lines. The Sankey diagram was generated by R v.4.1.2. The final visualization was further refined using Adobe Illustrator v.24.0.

### Virulence genes

The virulence factor profiles varied among the 24 serotype-ST combinations (Fig. 4 and Table S3). Based on their distribution, virulence factors were classified as core (detected in ≥23 of 24 serotype-ST combinations), broadly distributed (8–22 combinations), restricted (2–7 combinations), or serotype-ST-specific (one combination). Core virulence factors included genes associated with adherence (*csg, fim*, and *ecp/yag*), antimicrobial activity/competitive advantage (*acrB*), effector delivery (*gsp*), invasion (*ibeBC* and *ompA*), immune modulation (*gndA*), nutrient acquisition and metabolism (*allB, ent, fep*, and *fes*), and regulation (*fur, phoP, pmrA*, and *rcsB*).

**Fig. 4:**
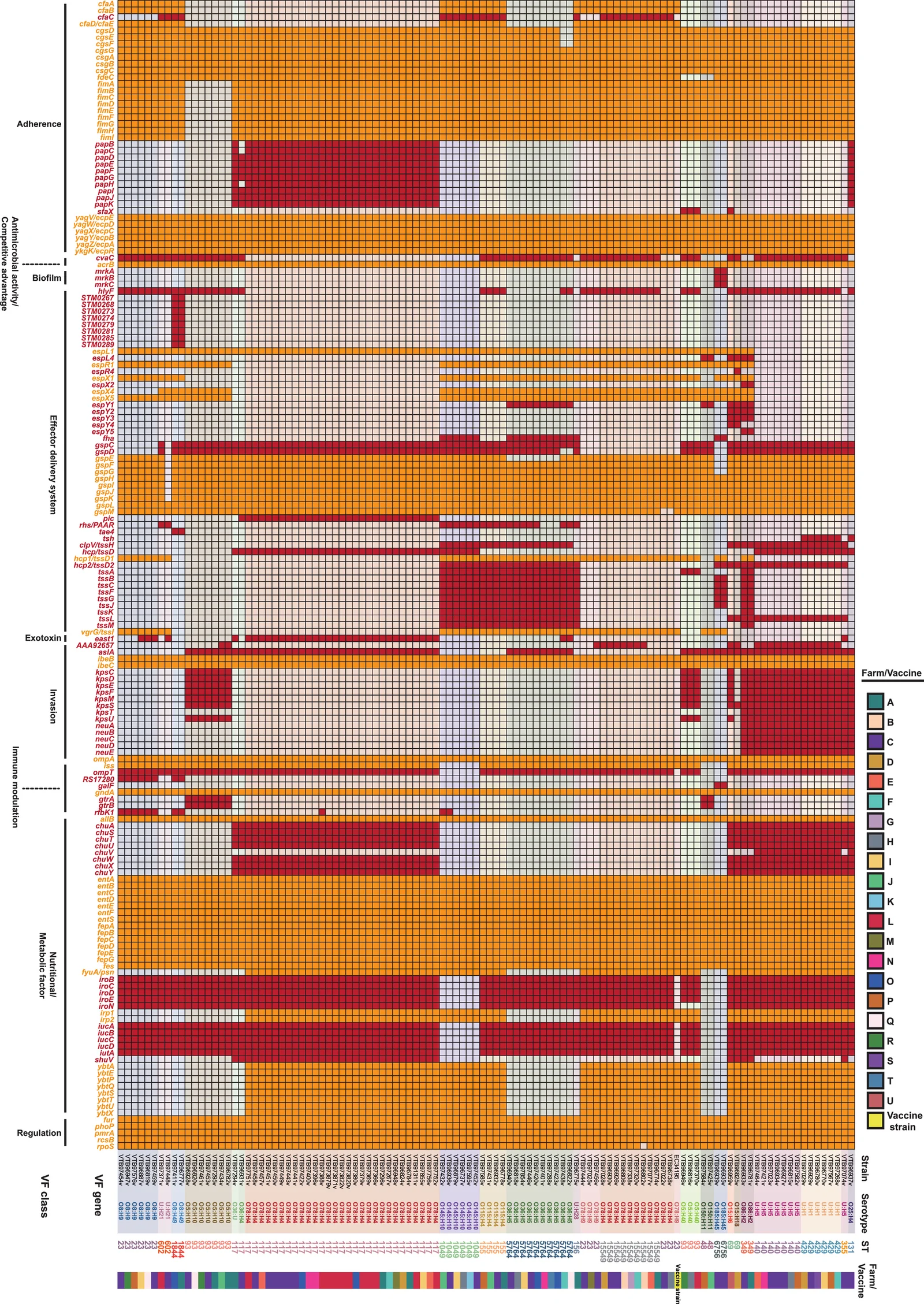
Distribution of virulence factors (VF) class and virulence-associated genes among 109 APEC strains recovered from poultry with airsacculitis in South Africa and vaccine strain EC34195. The matrix shows the presence and absence of virulence-associated genes across the strains. Genes detected in both vaccine strain EC34195 and field APEC strains are indicated in orange, whereas genes only detected in field strains are indicated in red. Strain names, serotype, sequence type (ST), and farm origin are indicated below the matrix. Virulence factors are grouped according to their functional classes. The matrix was generated by TBtools v.2.472, and strain information was annotated and visualization refined using Adobe Illustrator v.24.0.

Several other virulence factors were broadly distributed but varied among serotype-ST combinations, including *cfa, fdeC, tss, esp, aslA, kps, chu, fyuA/psn, irp, ybt, cvaC, hlyF, ompT, iss, iro*, and *iuc*. In contrast, *pap, sfaX, fha, pic, rhs/PAAR, tsh, east1, AAA92657, neu, KP1_RS17280/rfbK1, gtr*, and *shuV* showed more restricted distributions. A small number of virulence factors were confined to individual serotype-ST combinations, including *mrkABC* and *galF* in O185:H45-ST6756 and the STM genes and *tae4* in O8:H49-ST1844. The complete distribution of individual virulence factors across serotype-ST combinations is provided in Table S3.

Additionally, vaccine strain EC34195 (O78:H9-ST23) shared virulence genes with the investigated field strains with an overall detection rate of 45.9% (79/172) (marked in orange color in Fig. 4), indicating an overlap between virulence genes present in strain EC34195 and those detected in the field strains. However, strain EC34195 lacked other virulence clusters frequently occurring among the field strains like those related to iron transport (*iroBCDEN*) and aerobactin siderophore (*iutA-iucABCD*) systems, heme uptake and metabolism (*chuASTUWXY*), capsule synthesis (*kpsCDEFMSTU-neuABCDE*), secretion systems (*gsp, tsh, tssABCFGJKL*), and fimbriae (*papBCDEFGHIJK*) (marked with red color in Fig. 4).

### Antibiotic resistance and mechanisms

All 109 strains were phenotypically susceptible to meropenem, azithromycin, tigecycline, and colistin (Supplementary Table S4). Only 12.8% (14/109) of the strains were susceptible to all tested antimicrobials. The most frequent resistances consisted of those to nalidixic acid [71.6% (78/109)], tetracycline [71.6% (78/109)], trimethoprim [52.3% (57/109)], ampicillin [51.4% (56/109)], ciprofloxacin [50.5% (55/109)], sulfamethoxazole [41.3% (45/109)], and chloramphenicol [30.3% (33/109)]. Lower frequencies of resistance were observed for gentamicin [4.6% (5/109)], cefotaxime [3.7% (4/109)], and ceftazidime [0.9% (1/109)]. Multidrug resistance to 3 to 9 antibiotics was found in 58.72% (64/109) of the strains (Fig. 5 and Table S4). O78:H4-ST117 and O36:H5-ST5764 were the serotype-ST combinations that harbored the highest numbers of antimicrobial resistance mechanisms, with up to nine mechanisms detected (Fig. 5).

**Fig. 5:**
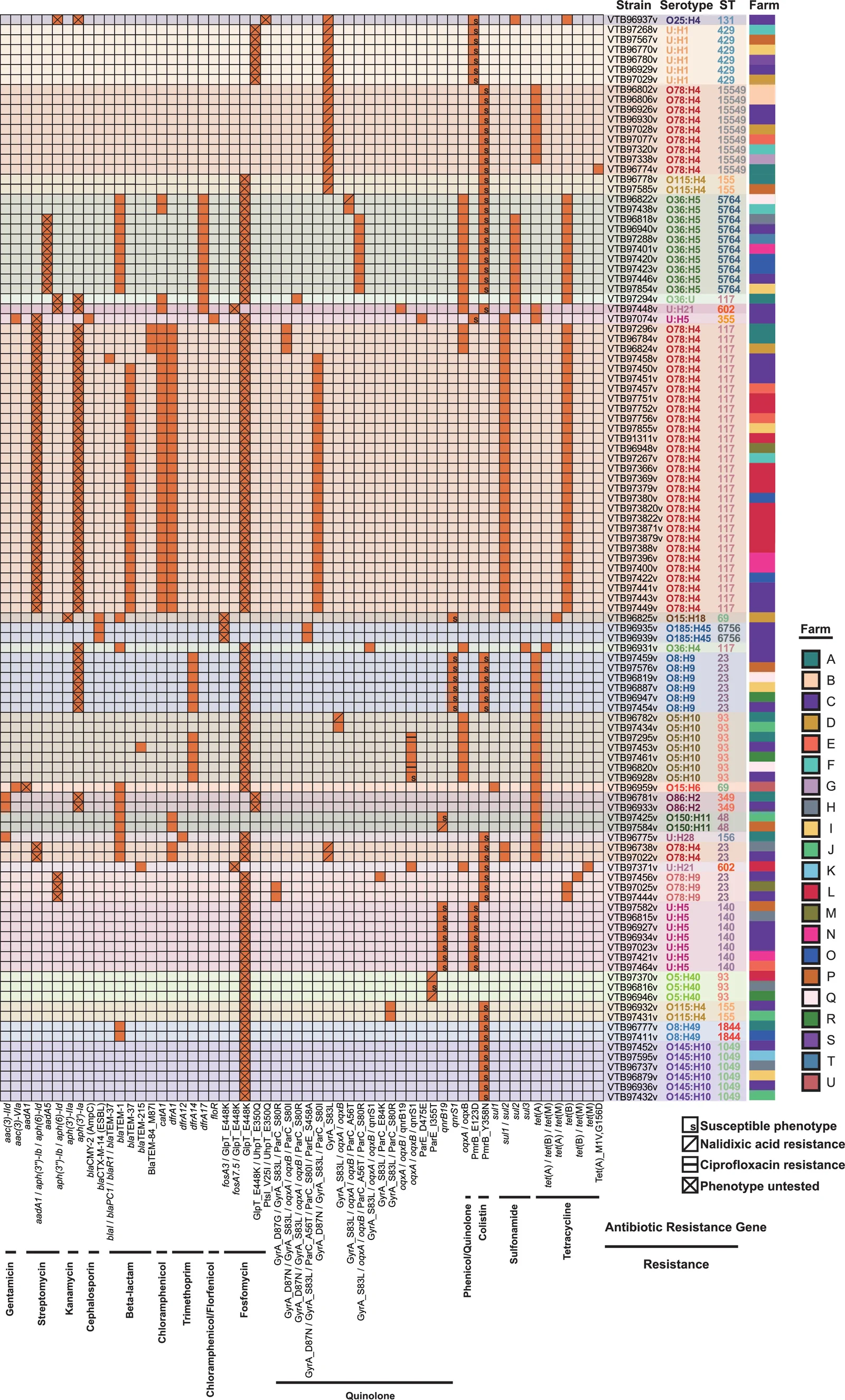
Distribution of antibiotic resistance determinants in 109 APEC strains from poultry airsacculitis in South Africa. Presence of a resistance mechanism is indicated by orange square. Associated phenotypes are indicated underneath the corresponding antimicrobial resistance mechanisms. Untested and specific phenotypes are indicated. Strain names, serotypes, sequence types (ST), and farms are indicated on the right of the matrix. The matrix in this figure is generated by TBtools v.2.472, and strain information was annotated and visualized using Adobe Illustrator v.24.0.

An association between phenotypic resistance and the presence of an antimicrobial resistance mechanism was observed for most of the strains (Fig. 6). Some of the strains remained susceptible to nalidixic acid and ciprofloxacin despite the presence of *qnrS1, qnrB19*, or *oqxAB.* Strains containing a single amino acid substitution in GyrA (S83L) were resistant to nalidixic acid and susceptible to ciprofloxacin as expected. Three strains containing the *catA1* gene and one strain containing *tet*(M) were susceptible to chloramphenicol and tetracycline, respectively.

**Fig. 6:**
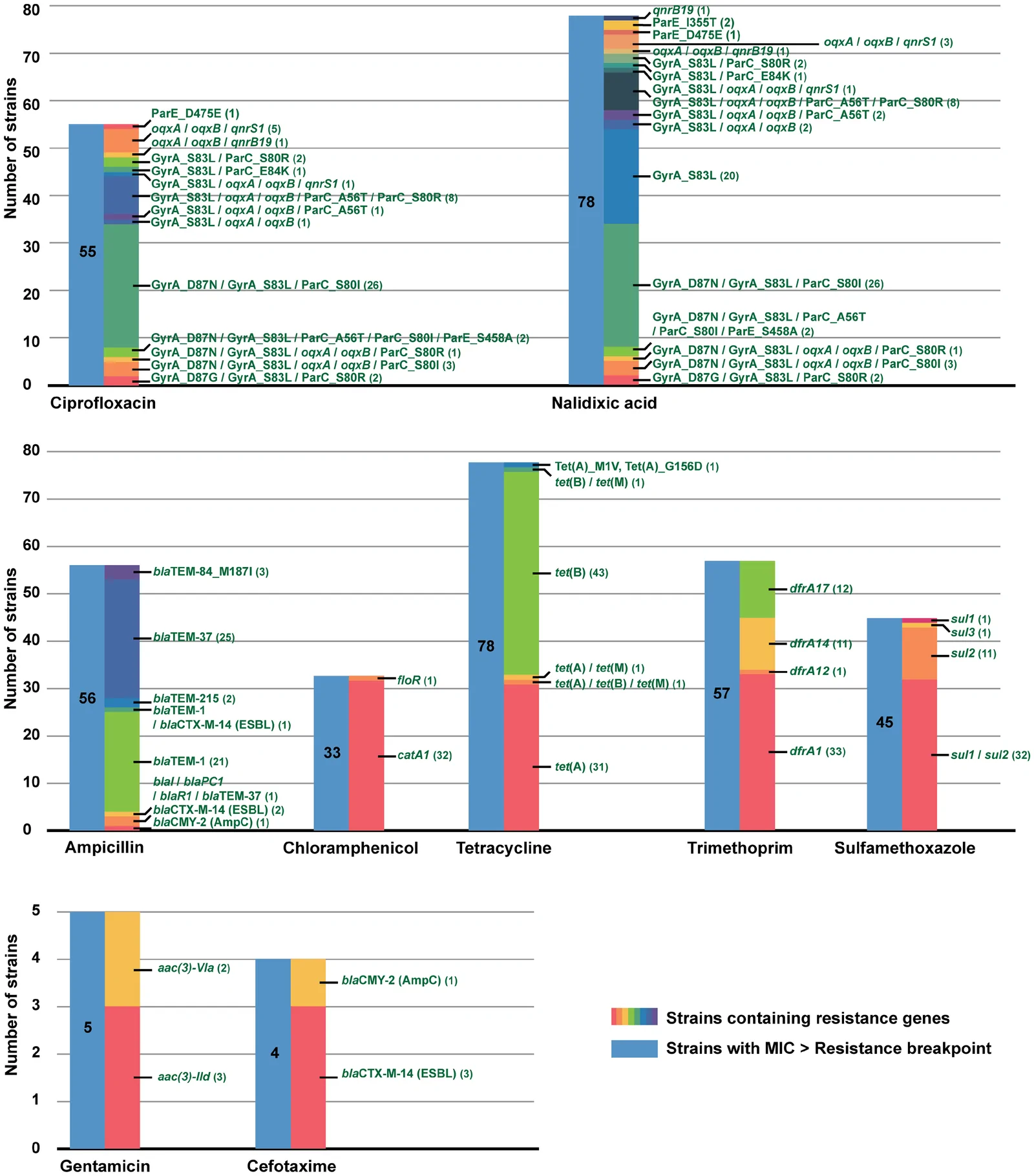
Distribution of genotypic and phenotypic antimicrobial resistance and corresponding resistance mechanisms among APEC strains from poultry with airsacculitis in South Africa. The blue bars indicate the number of strains exhibiting phenotypic resistance to each tested antimicrobial. Resistance was defined as a MIC above the resistance breakpoint. Multicolored stacked bars indicate the number of strains harboring the corresponding antimicrobial resistance genes and/or chromosomal mutations. Numbers within the blue bars indicate the total number of resistant strains for each antimicrobial. Resistance mechanisms and the number of strains carrying each mechanism or combination of mechanisms are indicated alongside the corresponding stacked bars. The stacked bar chart was generated using Microsoft Excel 2019 v.1808. The final visualization was further refined using Adobe Illustrator v.24.0.

## Discussion

Although serogroups O1, O2, and O78 have traditionally been reported as the most predominant among APEC, an increasing number of studies in Asia, Europe, and America revealed that the epidemiological trend may be shifting, characterized by a relative decline in O1 and O2 serogroups and the emergence of new ones (Ewers et al., 2004; Zhao et al., 2005; Ozawa et al., 2008; Ronco et al., 2017; Afayibo et al., 2022; Wang et al., 2022; Wilczyński et al., 2022). This has also been observed among the APEC from South Africa, where O78 was identified as the predominant serogroup representing 39.4% of the 109 APEC isolates, while O1 and O2 strains were not detected among the at least 12 other identified serogroups. On the other hand, the genetic diversity among APEC strains with same serogroup highlights that a combination of the different typing systems (WGS-based typing methods like MLST, serotyping and Clermont typing) is necessary for a more precise genetic characterization (Dziva et al., 2013; Mangiamele et al., 2013). Although a correlation between ST and serotype was frequently observed, the serotype–ST combination enabled differentiation between APEC strains sharing the same ST but belonging to different serogroups, as well as between strains sharing the same serogroup but belonging to different STs. Based on combined typing method, a further SNP-based analysis was also necessary to determine close genetic relatedness between APEC within the same serotype-ST combination and identify the presence of genetically closely related strains.

WGS-based analysis also enables better characterization and comparison of APEC strains from different countries and facilitates the identification of the most common lineages causing diseases (Ewers et al., 2004; Mehat et al., 2021). Several lineages identified in this study have also been previously reported. For instance, O78:H4-ST117-G, the predominant lineage identified in our study, has been recognized as a typical combination in poultry based on epidemiological data (Mehat et al., 2021). This lineage has been implicated in large outbreaks of colibacillosis in the Nordic broiler production during 2014 to 2016 (Ronco et al., 2017). Notably, ST117 is common among APEC isolates and has been sporadically observed prior to 2014 (Ronco et al., 2017). ST117 and ST140 are also known among pathogenic *E. coli* in humans (Jamali et al., 2024; Saidenberg et al., 2024). Subsequent epidemiologic studies have shown that ST117 belongs to the relatively newly defined phylogroup G (Clermont et al., 2019) and is capable of encoding multiple O-antigens beyond the commonly associated O78 (Kim et al., 2017), as was also observed for O78-ST117-G and O36-ST117-G in this study. On the other hand, the O78 serogroup is frequently represented by two distinct APEC lineages, ST23 in phylogroup C and ST117 in phylogroup G (Mehat et al., 2021). In this study, a novel ST15549, a *recA*-allele variant of ST23, was identified in association with O78:H4-ST15549-C in several poultry farms of South Africa.

In addition to the most predominant lineage of O78:H4-ST117-G, other serotype-ST combinations were also previously reported. For example, O5:H10-ST93 and O78:H4-ST23 were identified in Norway in March and June of 2019 (Kravik et al., 2023), and ST602 with an untypeable O antigen in Japan between April 2005 and March 2017 (Misumi et al., 2023). A previous phylogenomic analysis of a worldwide collection of 4,846 poultry *E. coli* revealed that ST155-B1 was common among poultry isolates from Africa (Foster-Nyarko et al., 2021), but only a few ST155-O115:H4-B1 were identified in South Africa in this study.

Serotype-ST combinations that differed from those previously described were observed in South Africa. For instance, O7:U-ST93 was reported in Brazil whereas ST93 was associated with O5:H40 and O5:H10 in South Africa (Maluta et al., 2014). O2/O50:H1-ST429 was predominantly reported in Norway between September 2018 and August 2019 (Kravik et al., 2023) carried different sero-antigens from those of the ST429-U:H1 strains identified in this study. A *purA*-allele variant of ST155 (ST1049) carried the same O-antigen O145 as that reported among predominant APEC in China (Wang et al., 2022). However, APEC O145 from China belonged to a different serotype-ST-phylogroup combination (O145:H21-ST2505-C) from that identified in South Africa (O145:H10-ST1049-B1) (Wang et al., 2022).

Although only a limited number of isolates belonged to ST131 (n=1) and ST69 (n=2), which are globally dominant human ExPEC lineages, their presence in poultry warrants continued attention. Additionally, ST117 and ST23, two predominant STs in this study, are also ranked among the top 20 ExPEC associated with human extraintestinal infections globally, highlighting the broad dissemination potential of these lineages (Manges et al., 2019). However, the virulence specificity and degree of relatedness between *E. coli* of these STs from humans and poultry needs to be further studied to better evaluate their zoonotic significance.

Several APEC lineages identified in different farms in this study were uncommon (O185:H45-ST6756-A, O36:H5-ST5764-A, and O8:H49-ST1844-B1) or so far unclassifiable by serogroup (U:H1-ST429-B2, U:H5-ST140-B2, U:H21-ST602-B1) highlighting that a large diversity of APEC can cause fatal infections in poultry and the difficulty of developing a broadly protective vaccine (Mehat et al., 2021). A vaccine with broader protection, guided by timely molecular epidemiological surveillance, would be an ideal strategy to control APEC infections.

Both cgMLST and cgSNP analyses among the isolates indicated the existence of genetically closely related strains across different farms in South Africa. Several strains belonging to the same serotype-ST combination differed from each other by less than 20 cgSNPs. For instance, the strains of the predominant lineage O78:H4-ST117-G differed only by 0–9 cgSNPs (Fig. 2). The presence of genetically closely related strains in both breeders and broilers within the same farms and cross different farms suggests possible horizontal and vertical transmission of strains within the poultry production system (Fig. 3). Such dissemination could potentially be associated with the movement of fertile eggs and newly hatched chicks (Joseph et al., 2023).

All 10 key virulence-associated factors recently highlighted in APEC (*iss, tsh, iroN, ompT, iutA, cvaC, hlyF, iucD, papG*, and *papC*) (Ovi et al., 2023) were identified in this study. These genes are associated with iron acquisition and siderophore systems (*iroN, iutA, iucD*), as well as *papG, papC* (pyelonephritis-associated pilli), *tsh* (TSH autotransporter secretion), *iss* (serum resistance factor), *ompT* (outer membrane protein T), *cvaC* (colicin V operon), and *hlyF* (promotes outer membrane vesicle biogenesis). Most of these genes are regarded as markers of ColV-like plasmids (ColVLPs) (Lian et al., 2025), which play an important role in the dissemination of virulence among the ExPEC population (Reid et al., 2022; Lian et al., 2025). Additional core and broadly distributed virulence genes, *fim, ompA, ibeBC, fep, iro, iuc, cfa, fdeC, esp, aslA, kps, chu, fyuA/psn, irp*, and *ybt* were frequently observed in association with the virulence of APEC strains (Ewers et al., 2007; Bonnet et al., 2009; Krishnan et al., 2015; Silveira et al., 2016; Hu et al., 2022; Ovi et al., 2023; Nawaz et al., 2024; Kerek et al., 2025). The *east1* gene, which encodes the enteroaggregative *E. coli* heat-stable enterotoxin and is commonly detected in diarrheagenic *E. coli* in humans (Savarino et al., 1991; Zhang et al., 2016), was detected in the predominant lineage of O78:H4-ST117 as well as in O36:H5-ST5764, O36:U-ST117, O8:H9-ST23, U:H21-ST602, and U:H5-ST355. This gene has already been reported in APEC at varying prevalence rates, suggesting that APEC and other pathogenic intestinal *E. coli* may share common virulence factors (Subedi et al., 2018; Al-Kandari and Woodward, 2019). Additionally, several genes were detected with relatively higher frequency in the prevalent strains belonging to O78:H4-ST117-G, including the *pap* gene cluster (*papBCDEFGHIJK*) and *pic*, which are both responsible for bacterial adhesion (Ovi et al., 2023). Overall, the virulence factor profiles varied across lineages, with clear phylogenetic and genotypic differences observed between vaccine strain EC34195 and field isolates (Fig. 4). Strain EC34195 lacked several important APEC-associated virulence operons like those related to iron transport, aerobactin siderophore systems, heme uptake and metabolism, capsule synthesis, secretion systems, and fimbriae. These differences indicate distinct virulence gene repertoires between vaccine strain EC34195 and field strains. However, whether these genotypic differences would influence vaccination protection or result in vaccine-driven selection cannot be determined from the present data and requires further phenotypic and experimental study.

Antimicrobial resistance gene detection and MIC testing indicated that multidrug resistance was widespread in APEC causing airsacculitis in poultry in South Africa with 58.7% of the 109 strains exhibiting resistance to 3–9 antimicrobials. Resistance to quinolones, tetracyclines, penicillins, sulfonamides and chloramphenicol was the most frequent (Fig. 6 and Table S4) as already observed in previous studies (Yang et al., 2004; Wang et al., 2010; Thomrongsuwannakij et al., 2020; Goudarztalejerdi et al., 2022). Resistance to the third generation cephalosporins was detected in only a few strains, and no antimicrobial resistance was detected to azithromycin, colistin, meropenem, or tigecycline, which are considered critically important antimicrobials for human medicine (Amoako et al., 2020). Although mutations within the *pmrAB* genes which activate the two-component system PmrAB are one of the primary colistin resistance mechanisms in *E. coli* (Huang et al., 2020), the E123D and Y358N mutations detected in PmrB in this study were not associated with phenotypic colistin resistance (Table S4). Additionally, none of the strains carried the colistin resistance gene *mcr*, which was associated with mobile genetic elements (Liu et al., 2016). This gene was previously reported in APEC in South Africa and in the meantime, the use of colistin has been banned (Perreten et al., 2016). The detection of a single amino acid substitution in GyrA (S83L), associated with nalidixic acid resistance (Table S4), indicates an early stage in the development of ciprofloxacin resistance and highlights the need for prudent use of second-generation quinolones (Kalinen et al., 2025). A few strains harboring *qnrS1* and *qnrB19* did not exhibit resistance to quinolones. These plasmid‑mediated quinolone resistance (PMQR) genes that encode Qnr‑like proteins protecting DNA gyrase and topoisomerase IV from fluoroquinolones generally confer only low-level to moderate fluoroquinolone resistance when present alone (Jacoby et al., 2014).

Overall, this study revealed genomic evidence consistent with inter- and intra-farm dissemination. Specific virulence factors and antibiotic resistance may facilitate the adaptation in the poultry niches and collectively explain the spread of APEC. Continuous interactions of APEC with various environments may enable them to adapt to new niches by acquiring new traits, giving them advantages to overcome antimicrobial and vaccination. These factors may contribute to the acceleration of evolution and diversification of APEC, resulting in diverse populations across different times and locations. With the development of sequencing technology, traditional typing methods may provide insufficient resolution for detailed epidemiological tracking. A comprehensive understanding of APEC epidemiology benefits from combining multiple WGS-based typing systems.

## Author contributions

**Zhuohao Wang** contributed to the conceptualization of the study, wrote the draft manuscript, conducted data analysis, performed software-based analyses, and drew the figures. **Naima A. Guthauser** performed antimicrobial susceptibility testing, conducted antimicrobial resistance data analysis, designed the phylogenetic figure. **David Gerber** designed and conceptualized the study, coordinated and supervised sampling and isolation of bacterial isolates. **Vincent Perreten** designed and conceptualized the study, coordinated and supervised the project, obtained financial support, edited and revised the manuscript. All authors have reviewed and approved the final version of the manuscript, and agree to be accountable for all aspects of the work.

## Funding information

This research was funded by internal funds (REF-660-50) of the Institute of Veterinary Bacteriology, University of Bern, Switzerland to V.P.

## Ethical statement

The samples were collected from animal carcasses; therefore, ethical approval was not required.

## Disclosures

The authors declare no conflicts of interest.

## Footnotes

Supplementary material associated with this article can be found, in the online version, at doi:10.1016/j.psj.2026.107885.
